# Appropriateness of antibiotic treatment of acute respiratory tract infections in Tunisian primary care and emergency departments: a multicenter cross-sectional study

**DOI:** 10.1186/s12875-022-01904-7

**Published:** 2022-11-22

**Authors:** Khaoula Bel Haj Ali, Adel Sekma, Selma Messous, Imen Trabelsi, Jalel Ben Youssef, Hamida Maghraoui, Rabie Razgallah, Adel walha, Mohamed Habib Grissa, Kaouthar Beltaief, Zied Mezgar, Ahmed Coubantini, Wahid Bouida, Mohamed Amine Msolli, Riadh Boukef, Hamdi Boubaker, Semir Nouira

**Affiliations:** 1 Emergency Department, Fattouma Bourguiba University Hospital, 5000 Monastir, Tunisia; 2 Research Laboratory LR12SP18, Monastir University, 5019 Monastir, Tunisia; 3 Vice-president of the Tunisian Society of Family Medicine, Tunis, Tunisia; 4 Emergency Department, Rabta University Hospital, 1007 Tunis, Tunisia; 5 DACIMA Consulting, 1053 Tunis, Tunisia; 6 Emergency Department, Farhat Hached University Hospital, 4031 Sousse, Tunisia; 7 Department of Infectious Disease, Rabta University Hospital, 1007 Tunis, Tunisia; 8 Emergency Department, Sahloul University Hospital, 4011 Sousse, Tunisia

**Keywords:** Acute respiratory tract infections, Antibiotics, Appropriateness

## Abstract

**Background:**

Little is known about the pattern and appropriateness of antibiotic prescriptions in patients with acute respiratory tract infections (ARTIs).

**Objective:**

Describe the antibiotics used to treat ARTIs in Tunisian primary care offices and emergency departments (EDs), and assess the appropriateness of their use.

**Methods:**

It was a prospective multicenter cross-sectional observational clinical study conducted at 63 primary care offices and 6 EDS during a period of 8 months. Appropriateness of antibiotic prescription was evaluated by trained physicians using the medication appropriateness index (MAI). The MAI ratings generated a weighted score of 0 to 18 with higher scores indicating low appropriateness. The study was conducted in accordance with the Declaration of Helsinki and national and institutional standards. The study was approved by the Ethics committee of Monastir Medical Faculty.

**Results:**

From the 12,880 patients screened we included 9886 patients. The mean age was 47.4, and 55.4% were men. The most frequent diagnosis of ARTI was were acute bronchitis (45.3%), COPD exacerbation (16.3%), tonsillitis (14.6%), rhinopharyngitis (12.2%) and sinusitis (11.5%). The most prescribed classes of antibiotics were penicillins (58.3%), fluoroquinolones (17.6%), and macrolides (16.9%). Antibiotic therapy was inappropriate in 75.5% of patients of whom 65.2% had bronchitis. 65% of patients had one or more antibiotic prescribing inappropriateness criteria as assessed by the MAI. The most frequently rated criteria were with expensiveness (75.8%) and indication (40%). Amoxicillin-clavulanic acid and levofloxacin were the most inappropriately prescribed antibiotics. History of cardiac ischemia ([OR] 3.66; 95% [CI] 2.17–10.26; *p* < 0.001), asthma ([OR] 3.29, 95% [CI] 1.77–6.13; *p* < 0.001), diabetes ([OR] 2.09, 95% [CI] 1.54–2.97; *p* = 0.003), history of COPD ([OR] 1.75, 95% [CI] 1.43–2.15; *p* < 0.001) and age > 65 years (Odds Ratio [OR] 1.35, 95% confidence interval [CI] 1.16–1.58; *p* < 0.001) were associated with a higher likelihood of inappropriate prescribing.

**Conclusion:**

Our findings indicate a high inappropriate use of antibiotics in ARTIs treated in in primary care and EDs. This was mostly related to antibiotic prescription in acute bronchitis and overuse of expensive broad spectrum antibiotics. Future interventions to improve antibiotic prescribing in primary care and EDs is needed.

**Trial registration:**

the trial is registered at Clinicaltrials.gov registry (NCT04482231).

## Introduction

Respiratory tract infections (RTIs) are the most common reason for antibiotic prescription in primary care [1, 2]. Although current guidelines recommend restrictive use of antibiotics for upper and lower RTIs, there is a clear evidence that they are heavily overprescribed [3–6]. In United States, it was estimated that unnecessary and guideline-discordant antibiotic prescribing for acute respiratory tract infections (ARTIs) ranged from 50 to 75% in primary care [7, 8]. In emergency departments (EDs) where ARTIs account for substantial attendances, almost half of the antibiotics prescribed were inappropriate [9]. In addition to the unnecessary costs, antibiotics overuse may lead to further increase in drug resistance and side effects [10, 11]. While most of available studies on antibiotic utilization patterns in ARTIs were from European and North American populations [5, 12, 13], data from less developed countries with different populations characteristics and medical practice are lacking. Importantly, overprescribing of antibiotics for ARTIs are less acceptable in low-income countries where resources are highly constrained and optimization of limited health care facilities is even more essential [14]. Thus, specific studies are required to investigate overall antibiotic prescribing in such setting and to better inform antimicrobial stewardship. The present study describes the characteristics of patients consulting in Tunisian primary care offices and EDs treated with antibiotics for ARTIs and, more specifically, examines the appropriateness of antibiotic prescribing.

## Materials and methods

This is an observational, cross-sectional, multicenter, national clinical study. The study was carried out from January 2018 to August 2018 in Tunisian population involving 63 primary care outpatient offices (100 General/Family Practice physicians) and 6 EDs. The sampling was planned to cover most of Tunisian areas. In total, 20 counties were selected to reflect the national picture of antibiotic use.

### Search strategy

We performed an exhaustive search by consulting the different available sources as Medline (PubMed), Embase (Ovid), Global Health (Ovid), and CENTRAL (Cochrane Library) of studies conducted in primary care or in the emergency departments to estimate the prevalence of antibiotic prescriptions and first choice antibiotics for ARTIs. The search strategy was built using key terms for “antibiotic,” “primary healthcare,” “emergency”, “prescribing,” and “acute respiratory tract infections”. Bibliographies of retrieved articles were also searched for further studies, and we consulted the annual Tunisian health ministry reports.

### Ethics

The study was conducted in accordance with the Declaration of Helsinki and national and institutional standards. The study was approved by the Ethics committee of Monastir Medical Faculty and is registered at Clinicaltrials.gov registry (NCT04482231). We obtained free and informed consent of all included patients.

### Study population

We included patients over the age of 18 years presenting to the EDs or to primary care offices and received antibiotic treatment for lower or upper ARTIs, according to the International Classification of Primary Care. Lower ARTIs include pneumonia and acute bronchitis. Acute upper ARTIs include rhinitis, pharyngitis/tonsillitis, sinusitis, and laryngitis. Each patient was included in the study only once and only antibiotics for oral systemic use were recorded. We excluded any visit that resulted in admission to the hospital, patients with additional diagnoses requiring antibiotherapy, patients with history of immunodeficiency (e.g., systemic corticosteroid use, HIV positive) or active pulmonary tuberculosis. Patients who received antibiotics or who were discharged from the hospital within the preceding two weeks were also excluded. There were no standard antimicrobial order sets at the participating sites during the time of this study.

### Study protocol

For each patient, the general practitioner or EDs physician registered baseline demographics including age, sex, race, body weight, smoking status, diagnosis of ARTI type, symptoms, duration of symptoms and which antibiotics were prescribed. Additional data collected included comorbid conditions, including heart failure (HF), chronic obstructive pulmonary disease (COPD), asthma, and diabetes. We used the medication appropriateness index (MAI) [15, 16] which includes 10 different areas of medication prescribing (Table 1). Two blinded and experienced evaluators were involved separately in the appropriateness rating using MAI on the basis of local recommendations compiled from national and international guidelines [17, 18](Table 2). These guidelines were not available to Tunisian doctors at the time when data were collected. When a rating inconsistency was found, the agreement was reached by consensus by the evaluators.. For each criterion, the evaluator rates whether the medication is appropriate, marginally appropriate, or inappropriate. Support is provided to all participating assessors through explicit definitions and instructions to calculate MAI score. Ratings of clearly appropriate and marginally appropriate received no score. Weighted scores were assigned to clearly inappropriate ratings as shown in Table 1. The score for each antibiotic prescribed ranges from 0 to 18. A higher score indicates a greater degree of medication inappropriateness. If a patient was prescribed more than one antibiotic, this test was considered for only one (having the highest MAI). For the first 300 prescriptions (2.3% of the targeted sample size), two blinded investigators conducted a blinded independent double assessment of the MAI to check inter-rater reliability. Assessments on the appropriateness of therapy were made with reference to NICE guidelines [17, 18]. No specific treatment or intervention was planned in the management of the included patients. For data collection we used an online data collection electronic database (DACIMA Clinical Suite® in accordance with FDA 21 CFR part 11, HIPAA & ICH).

**Table1:** The medication appropriateness index criterion

	**Yes**	**No**
Is there an indication for the drug	0	3
Is the medication effective for the condition	0	3
Is the dosage correct	0	2
Are the directions correct	0	1
Are the directions practical^a^	0	1
Are there clinically significant drug-drug interaction	2	0
Are there clinically significant drug-disease/condition interactions	2	0
Is there unnecessary duplication with other drug(s)	1	0
Is the duration of therapy acceptable	0	1
Is this drug the least expensive alternative compared to others of equal utility	0	1

^a^ They included time of intake in relation to the meal, pharmaceutical form (tablet, syrup, etc.), dose, duration, precautions to take, and non-refundable mention when this is the case

**Table 2:** First choice antibiotics for RTIs according to local recommendations compiled from national and international guidelines

Pathologies	First choice antibiotic	Doses	Duration of treatment	Comments
Nasopharyngitis and tonsillitis	First choice For individuals without penicillin allergy			Prescribe antipyretics and analgesics.-*Lactam antibiotics are indicated according to FeverPAIN and/or Centor score*
	Penicillin V, oral	500 mg 4 times daily or 1000 mg twice daily	5 to 7 days	
	Amoxicillin, oral	500 mg twice Daily	5 to 7 days	
	Benzathine penicillin G, intramuscular	1 200 000 U	1 dose	
	Alternative first choice for penicillin allergy or intolerance			
	Clarithromycin, oral	250 mg twice daily	5 to 7 days	
	Azithromycin, oral	500 mg once daily	3 days	
Acute bronchitis	Never indicated	-	-	In the absence of pneumonia, antibiotics are not indicated. Routine testing for nonviral causes is not recommended
Sinusitis	First choice			Acetaminophen or ibuprofen can relieve pain and fever. Saline irrigations, or washing out the nose with salt water, can relieve symptoms and remove mucus that is hard to blow out. Nasal steroid sprays can reduce symptoms after 15 days of use. Antibiotics may be prescribed if symptoms last > 10 d, severe symptoms last for > 3 consecutive days, or worsening symptoms last after 3 consecutive days
	Penicillin V, oral	500 mg 4 times daily	5 days	
	Amoxicillin-clavulanate, oral	500/125 mg 3 times a day	5 to 7 days	
	Alternative first choices for penicillin allergy or intolerance			
	Doxycycline, oral	200 mg on first day, then 100 mg once a day	5 days	
	Clarithromycin, oral	500 mg twice daily	5 days	
Otitis media	First choice			Offer regular doses of paracetamol or ibuprofen for pain. Consider eardrops containing an anaesthetic and an analgesic for pain if an immediate antibiotic is not given, and there is no eardrum perforation or otorrhoea
	Amoxicillin, oral	500 mg three times a day	5 to 7 days	
	Amoxicillin-clavulanate, oral	500/125 mg 3 times a day	5 to 7 days	
	Alternative first choice for penicillin allergy or intolerance			
	Clarithromycin, oral	500 mg twice daily	5 to 7 days	
	Cefuroxime, oral	(30 mg/kg) per day in 2 divided doses	5 to 7 days	
Acute COPD exacerbation	First choice			Indication for antibiotic treatment of acute exacerbations of COPD - Severe or very severe COPD with purulent sputum - Mild and moderate COPD with purulent sputum and inflammatory syndrome (CRP > 40 mg/dl) - Mild and moderate COPD with purulent sputum that does not improve after 3 days of treatment with bronchodilator and physiotherapy
	Amoxicillin, oral	500 mg three times a day	5 to 7 days	
	Doxycycline	200 mg on first day, then 100 mg once a day	5 to 7 days	
	Azithromycin, oral	500 mg once daily	3 days	
	Clarithromycin	500 mg twice a day	5 to 7 days	

*Abbreviations*: *ARTIs* Acute respiratory tract infections, *COPD* Chronic obstructive pulmonary disease

### Statistical analysis

Qualitative variables were expressed as frequencies and percentage. Continuous variables were presented as means ± standard deviations or median and interquartile range as appropriate. We calculated the mean MAI for each antibiotic class and ARTI type. The normality of the continuous quantitative variables was verified with the Shapiro–Wilk test. To identify factors associated with inappropriate prescription we tested the univariable relationship between the independent variables for inappropriate prescribing of antibiotics using logistic regression. Those that were significant at an alpha of 0.1 or less were included in a multivariable logistic regression model. Independent variables were demographic characteristics including gender, age, comorbidities, and clinical variables. A *p* value < 0.05 was considered a level of statistical significance. Data were analyzed using SPSS version 20 (SPSS Inc, Chicago, IL).

## Results

**Fig. 1:**
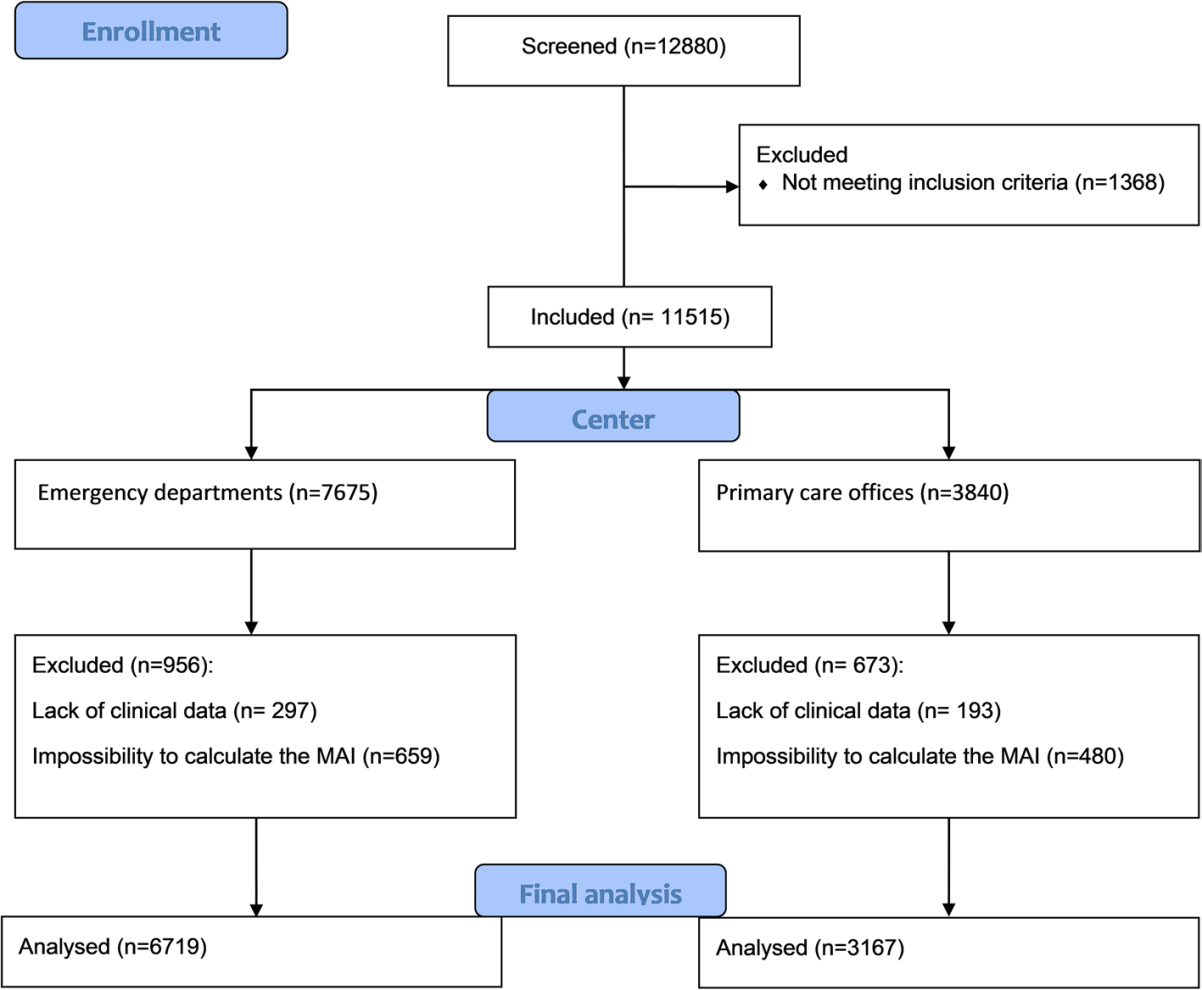
Flow chart of patients’ selection

**Fig. 2:**
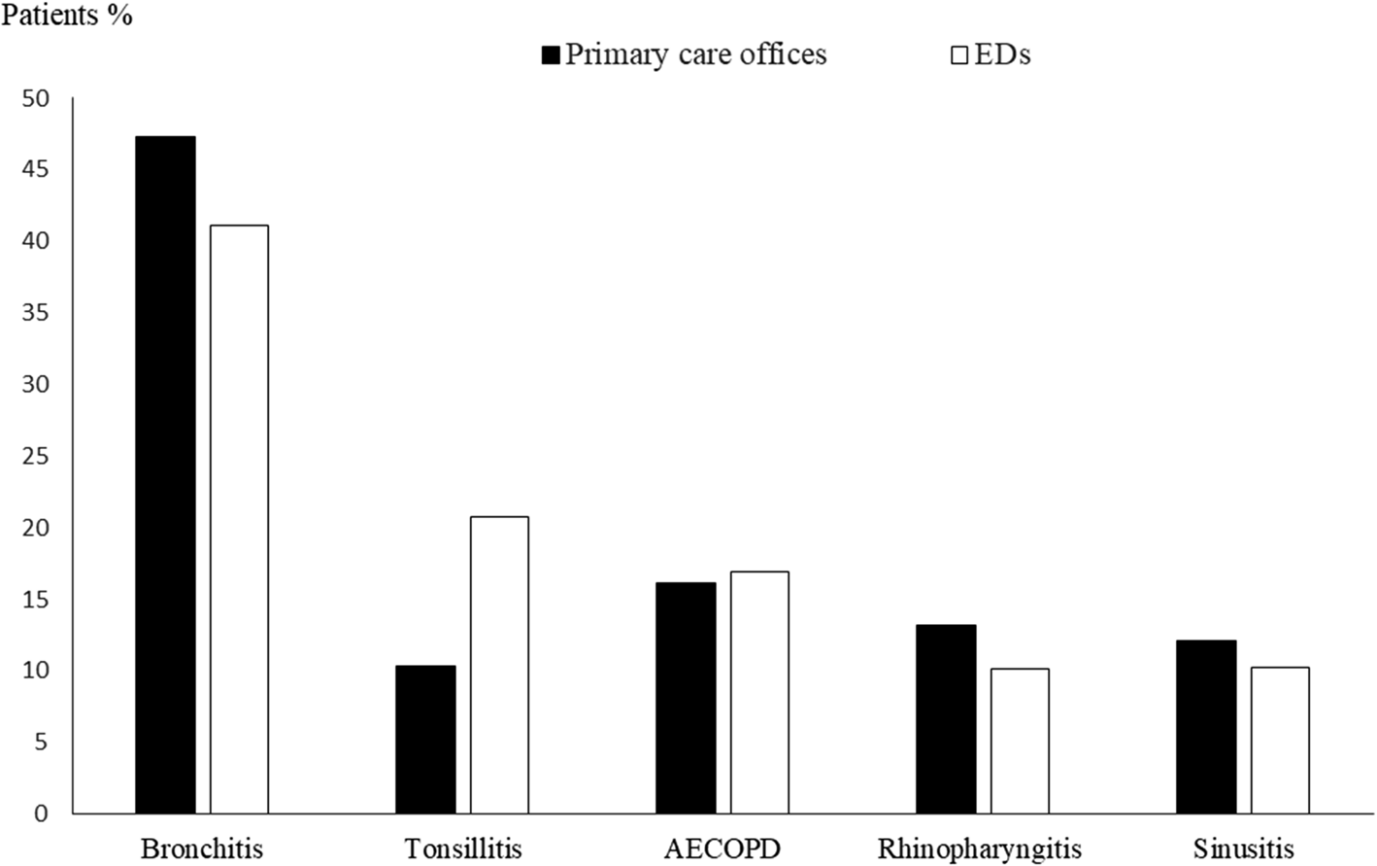
The leading diagnosis accounting for antibiotic prescriptions in primary care offices and emergency departments. Abbreviations: EDs, Emergency Departments, AECOPD, acute exacerbation of chronic obstructive pulmonary disease

**Fig. 3:**
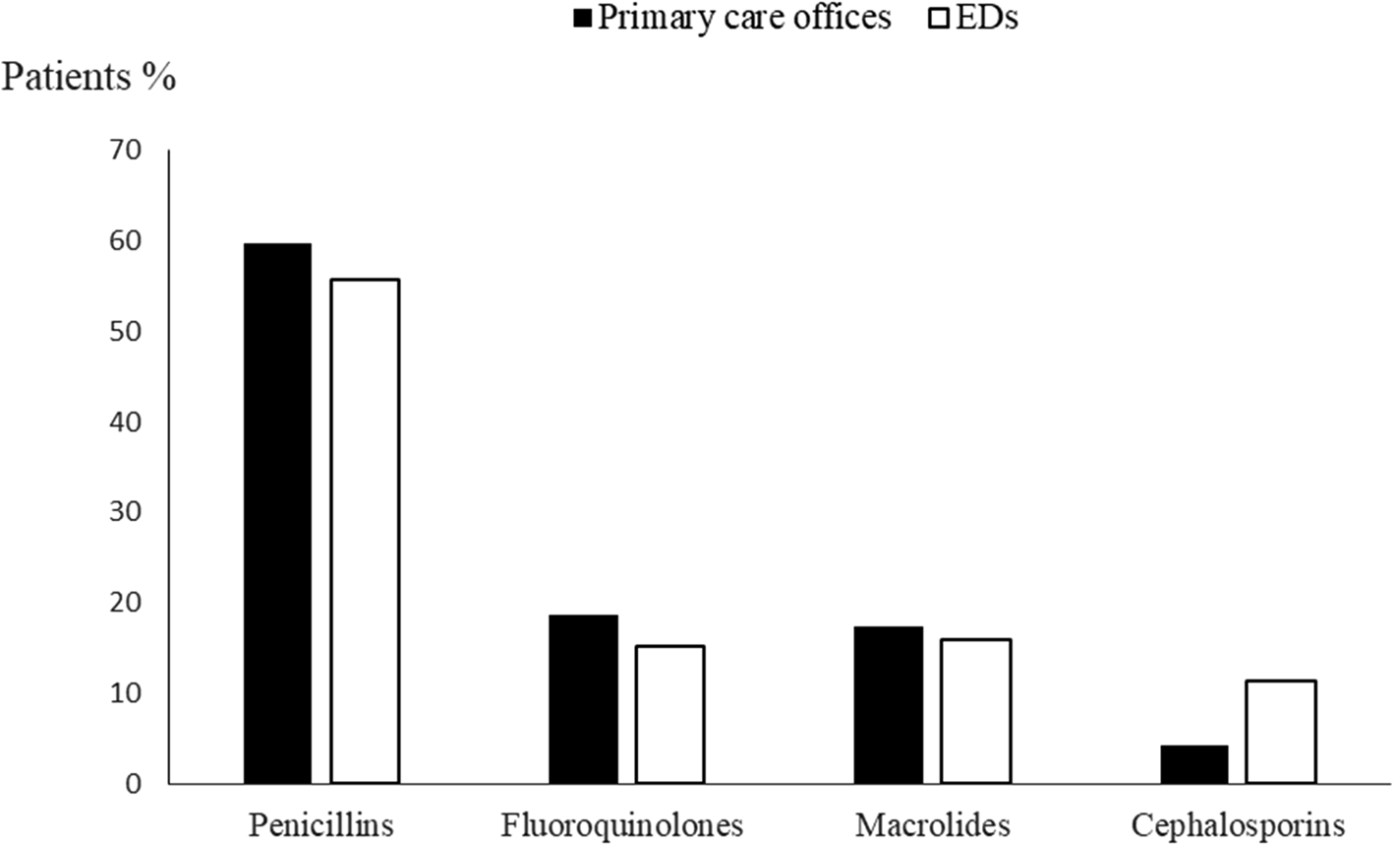
The most prescribed antibiotics in primary care offices and emergency departments. Abbreviations: EDs, Emergency Departments

We screened 12,880 patients and we included 9886 patients, 6719 from primary care offices and 3167 from EDs. 2994 patients were excluded for the following reasons: predefined exclusion criteria (*n* = 1365), lack of clinical data (*n* = 490), and impossibility to calculate the medication appropriateness index (*n* = 1139) (Fig. 1). Mean age was 47.4 ± 18 years and 55% were male. The most reported comorbidities were arterial hypertension (20.7%), diabetes (17.2%) and active smoking (21.7%). Mean time between the onset of symptoms and the day of consultation was 2.3 days. Cough (60.3%), sputum (36.6%) and runny nose (26.5%) were the most common symptoms (Table 3). The largest number of prescriptions was provided by primary care physicians, accounting for 67.9% of total prescriptions. The leading diagnoses accounting for antibiotic prescriptions in the overall population were acute bronchitis (45.3%), COPD exacerbation (16.3%), tonsillitis (14.6%), rhinopharyngitis (12.2%) and sinusitis (11.5%). There was no significant difference between primary care and ED antibiotic prescriptions with regard to ARTIs distribution (Fig. 2). The most prescribed classes of antibiotics were penicillins (58.3%), fluoroquinolones (17.6%), macrolides (16.9%), and cephalosporins (6.5%) (Fig. 3). There was no significant difference between primary care offices and EDs prescriptions with regard to the antibiotics used. Amoxicillin-clavulanic acid (48.7%), amoxicillin (13.7%), levofloxacin (12.5%), cefixime (9.2%), ciprofloxacin (8.6%), and azithromycin (3.3%) were the most commonly prescribed antibiotics.

**Table 3:** Patients’ baseline characteristics

	**Overall** *n* = 9886	**Primary care offices** *n* = 6719	**EDs** *n* = 3167
**Age, mean ± SD**	47.4 ± 18	50.2 ± 12.3	47.7 ± 16.8
**Sex-ratio (M/F)**	1.23	2.29	2.14
**Active smoking, n (%)**	2148 (21.7)	1559 (23.2)	589 (18.6)
**Past medical history, n (%)**			
Diabetes	1697 (17.2)	1095 (16.3)	602 (19)
Hypertension	2048 (20.7)	1424 (21.2)	624 (19.7)
COPD	1805 (18.2)	1165 (17.3)	640 (20.2)
**Symptoms, n (%)**			
Cough	5959 (60.3)	3783 (56.3)	2176 (68.7)
Sputum	3626 (36.6)	2258 (33.6)	1368 (43.2)
Runny nose	2624 (26.5)	1807 (26.9)	817 (25.8)
Sore throat	2186 (22.1)	1176 (17.5)	1010 (31.9)
Headache	2011 (20.3)	1283 (19.1)	728 (23)
Dysphagia	1929 (19.5)	1337 (19.9)	592 (18.7)
Fever	1925 (19.5)	1384 (20.6)	541 (17.1)

*Abbreviations*: *EDs* Emergency Departments, *COPD* Chronic obstructive pulmonary disease

Of the total prescriptions included, 1621 (24.5%) received no inappropriate ratings, 62.1% had one, 10.3% had two, and 3.1% had three or more. Table 4 shows the MAI ratings by prescribing criteria. Inappropriate ratings were less frequent for drug-disease interactions (4.4%), drug-drug interactions (4%) and therapeutic duplication (3.7%). The percentage of inappropriate ratings was higher for cost (75.8%) and indication (40%). The mean MAI score per antibiotic was 9.2 ± 1.3. Table 5 shows mean scores by antibiotic for the most prescribed ones. The MAI score ranged from 4.2 ± 0.8 for COPD exacerbation to 12.8 ± 5.3 for bronchitis. MAI score was lowest when azithromycin and cefuroxime were prescribed (2.1 ± 2.6 and 4.5 ± 3.4 respectively). The factors that were associated with inappropriate antibiotic prescribing were history of cardiac ischemia ([OR] 3.66; 95% [CI] 2.17–10.26; *p* < 0.001), asthma ([OR] 3.29, 95% [CI] 1.77–6.13; *p* < 0.001), diabetes ([OR] 2.09, 95% [CI] 1.54–2.97; *p* = 0.003), history of COPD ([OR] 1.75, 95% [CI] 1.43–2.15; *p* < 0.001) and age > 65 years (Odds Ratio [OR] 1.35, 95% confidence interval [CI] 1.16–1.58; *p* < 0.001).

**Table 4:** Proportions of inappropriate ratings for prescribing criteria of the Medication Appropriateness Index

Criteria	Inappropriate ratings, n (%)
Cost	5658 (75.8)
Indication	2986 (40)
Correct directions	1216 (16.3)
Medication effectiveness	1873 (16.1)
Practical directions	1104 (14.8)
Dosage	1104 (14.8)
Duration of treatment	784 (10.5)
Drug-drug interactions	328 (4.4)
Drug-disease interactions	379 (4)
Therapeutic duplication	276 (3.7)

**Table 5:** Mean Medication Appropriateness Index for the most frequent acute respiratory tract infections and antibiotics used

	**Medication Appropriateness Index**^**a**^ mean (SD)
**Antibiotic**	
Amoxicillin clavulanic acid	11.3 ± 2.8
Levofloxacin	10.3 ± 4.3
Amoxicillin	8.6 ± 3.3
Cefuroxime	4.5 ± 3.4
Azithromycin	2.1 ± 2.6
**Acute respiratory tract infection**	
Bronchitis	12.8 ± 5.3
Sinusitis	11.7 ± 4.3
Rhinopharyngitis	10.0 ± 1.5
Tonsillitis	9.3 ± 2.8
AECOPD exacerbations	4.2 ± 0.8

*Abbreviations*: *AECOPD* Acute exacerbation of chronic obstructive pulmonary disease

^a^ The Medication Appropriateness Index ranges from 0 to 18. A higher score indicates a greater degree of medication inappropriateness

## Discussion

### Main findings

Our study showed that most ARTIs treated with antibiotics in primary care and EDs were bronchitis, tonsillitis, COPD exacerbation, rhinopharyngitis and sinusitis. The most used classes of antibiotics were penicillins accounting for more than 58% of the total antibiotics prescribed for ARTIs. Among these, the most commonly prescribed penicillin was amoxicillin clavulanate followed by amoxicillin. Fluoroquinolones accounted for 17.6% of all antibiotic prescriptions, and 49% of these were levofloxacin. Macrolides and cephalosporins were far less frequently prescribed. In 75.8% of cases, antibiotic therapy should not be prescribed. Inappropriate antibiotic prescription as assessed by MAI was mostly observed in acute bronchitis and in patients treated with amoxicillin-clavulanic acid or levofloxacin. Comorbidities were significantly associated with inappropriate antibiotic prescription.

### Comparison with other studies

There is clear evidence that antibiotics are heavily overprescribed for respiratory infections because most of these infections are of viral origin and self-limited conditions [1, 3, 19]. Their prescription rate ranged between 20 and 90% in Europe [12, 20, 21] and 50 to 70% in United States [21]. Our study highlighted the worldwide variation in types of RTIs treated and patterns of antibiotics used. In a study conducted in the UK [22] targeting primary care settings, 73% of antibiotic prescriptions used in the treatment of upper respiratory tract infections were penicillins which is similar to our findings. According to a tertiary medical institution study conducted in Beijing [23], the most commonly prescribed classes of antibiotics for ARTIs were cephalosporins (41%). In Japan, cephalosporins constituted 41.9% of all antibiotic prescriptions and penicillins accounted for just 8.0% [24]. In our study, we noted a frequent use of broad-spectrum antibiotics, amoxicillin clavulanic acid and levofloxacin represented almost two thirds of all antibiotics prescribed. This practice is not appropriate as it is recommended that narrow-spectrum antibiotics should be maintained at ≥ 80% in cases prescribed an antibiotic, while the proportion of fluoroquinolones should be maintained at ≤ 5% [25, 26]. Overall, the quality of prescribing was inappropriate in our study as attested by MAI score. Similar results were observed in the United States and other developed countries [27–30]. The most common MAI item involved was expensiveness and indication while the antibiotics that were most often prescribed inappropriately were amoxicillin clavulanic acid and levofloxacin. In the last decade, one study was undertaken by the National Union of the Mutual Insurance Companies in Tunisia, with the approval of the Ministry for Public Health, it demonstrated that innovator brands were more widely used due to the promotional sales forces on the prescribers whereas the prices of innovator brands are considerably higher than the prices of Tunisian generic equivalents [31]. In our study, when the least expensive antibiotic is not prescribed, we considered that the decision was not appropriate. In countries with limited health resources, this indiscriminate use of antibiotics in ARTIs may result in increased health care cost. In the era of increased bacterial resistance, the need to restrict antibiotic prescription with special emphasis to narrow spectrum ones is more than urgent. Our study is the first to investigate physician practice in Tunisian EDs where the utilization rate of antibiotics for ARTIs could exceed the rate of ambulatory setting. High-volume workload, high-acuity nature of ED clinical presentation, and specificity of patient-physician relationships in the ED could explain why ED physician are more exposed to prescribe antibiotics inappropriately. In a study conducted in United States including ED visits with a diagnosis of ARTI, it was found that approximately 40% of antibiotic prescriptions were inappropriate [9]. Improving the appropriate use of antibiotics in ARTIs in primary care or EDs should take into account the factors that could be implicated in this phenomenon. Available data indicate the existence of a great variation between countries with regard to the factors associated with inappropriate antibiotic prescription [12, 32]. Patient expectation and physicians related factors such as diagnostic uncertainty, lack of awareness of specific guideline recommendations, and lack of time necessary to reassure the patient were among the principal reasons of antibiotic overprescription. Our study was focused on patients’ characteristics and we showed that history of coronary artery disease, asthma, and diabetes were the most important factors associated with antibiotherapy inappropriateness. Patients with diagnosis of acute bronchitis were also more likely to receive antibiotics inappropriately.

### Limits of the study

There are a number of potential limitations to note. First, although our study included a large sample representing overall clinical practice in Tunisia, we acknowledge that we did not include children who represent some of the highest users of antibiotic prescriptions. Second, in this study we applied the MAI score to assess prescribing appropriateness in primary care and ED practice. Whether this score is optimal when antibiotic inappropriateness is addressed is a question that should be clarified. Of note, this index is generally considered among the most acceptable available tools for implicit measurement of inappropriate prescribing. It was initially validated in geriatric outpatient population but has since been validated for use in inpatient settings. It was found to have good interrater and intrarater reliability. It has undergone extensive validity testing, in the USA, UK and, more recently, in Europe [33, 34]. MAI was shown to be a valuable tool for measuring potentially inappropriate prescribing for many types of medications; so there is no reason to exclude antibiotics from the MAI field of use in the absence of other evaluation scale. Third, it is possible that there is differences in antibacterial resistance patterns between UK and Tunisia; unfortunately, we have not available Tunisian data to objectively assess whether these differences exist and their potential impact in actual appropriateness of antibiotics’ choices. Finally, for some prescriptions classified as inappropriate, there could be individual patient factors unknown to reviewers that might justify a provider’s decision to deviate from the guidelines.

## Conclusion

Our study demonstrated that there is a high rate of inappropriate antibiotic prescribing for patients diagnosed with ARTIs in primary care and EDs. Incorrect indications such as acute bronchitis and choosing expensive and broad spectrum antibiotics were the most common reasons for inappropriate prescribing in particular for old patients with comorbid conditions. The potential for reducing rates of antibiotic prescription is therefore substantial. Future research should include interventions to improve the use of antibiotics in ARTIs.

## Data Availability

The datasets used and/or analysed during the current study are available from the corresponding author on reasonable request.

## Abbreviations

ARTIs: Acute respiratory tract infections
ED: Emergency department
MAI: Medication appropriateness index
COPD: Chronic obstructive pulmonary disease
HF: Heart failure
